# Impact of Educational Interventions on Acceptance and Uptake of Male Circumcision in the General Population of Western China: A Multicenter Cohort Study

**DOI:** 10.1038/s41598-017-13995-9

**Published:** 2017-11-02

**Authors:** Bo Zhou, Chuanyi Ning, Chase D. McCann, Yanyan Liao, Xiaobo Yang, Yunfeng Zou, Junjun Jiang, Bingyu Liang, Abu S. Abdullah, Bo Qin, Halmurat Upur, Chaohui Zhong, Li Ye, Hao Liang

**Affiliations:** 1 Guangxi Key Laboratory of AIDS Prevention and Treatment, School of Public Health, Guangxi Medical University, Guangxi, China; 2 Department of Microbiology & Immunology University of North Carolina at Chapel Hill, Chapel Hill, North Carolina USA; 3 Department of Medicine, Boston Medical Center, Boston University Medical Campus, Boston, United States of America; 4 The First Affiliated Hospital, Chongqing Medical University, Chongqing, China; 5 School of Public Health, Xinjiang Medical University, Xinjiang, China; 6 School of Public Health, Chongqing Medical University, Chongqing, China

## Abstract

To compare different intervention models for promoting male circumcision (MC) to prevent HIV transmission in Western China. A total of 1690 male participants from multiple study sites were cluster randomly allocated to three-stage (Model A), two-stage (Model B), and one-stage (Model C) educational interventions. In all three interventions models, knowledge about MC significantly increased and the reported willingness to accept MC increased to 52.6% (255/485), 67.0% (353/527), and 45.5% (219/481) after intervention, respectively (P < 0.05). Rate of MC surgery uptake was highest (23.7%; 115/485) among those who received Model A intervention, compared to those who received Model B (17.1%; 90/527) or Model C (9.4%; 45/481) interventions (P < 0.05). Multivariable Cox regression analysis identified that Model A or Model B had twice the effect of Model C on MC uptake, with relative risks of 2.4 (95%CI, 1.5–3.8) and 2.2 (95%CI, 1.3–3.6), respectively. Model B was the most effective model for improving participants’ willingness to accept MC, while Model A was most successful at increasing uptake of MC surgery. Self-reported attitude towards MC uptake was not strongly correlated with actual behavior in this study focusing on the general male population in Western China.

## Introduction

The most recent China AIDS Response Progress Report pointed out that heterosexual transmission accounted for more than 70% of all new HIV infections reported in China during 2016^1^. Additionally, epidemiological evidence has shown that the HIV/AIDS epidemic has expanded from high-risk groups to the general population, with a large increase of heterosexual transmission over the past decade^2–4^. The number of HIV infections in the Guangxi, Xinjiang and Chongqing Provinces of Western China alone, account for 27.3% of the total cases of HIV in China^5^. In addition, the proportion of men infected with HIV through sexual contact has increased from 7.3% in 2000 to 45.4% in 2009 in these areas, the number of HIV/AIDS cases caused by heterosexual transmission increased to 68,671 in 2014, accounted for 66.4%^6,7^. Extra-marital sex, multiple sex partners and high-risk sexual behaviors may contribute to these epidemiological changes witnessed in Western China in recent years^7,8^.

To battle this serious epidemic, a number of effective intervention strategies have been suggested, including male circumcision (MC)^9^. Multiple randomized controlled trials (RCTs) in Africa have demonstrated success of MC in preventing the acquisition of HIV by men through heterosexual intercourse^10–12^. Additionally, uncircumcised men have a significantly higher risk of acquiring other STIs including syphilis, high-risk human papillomavirus, herpes simplex type 2, and mycoplasma genitalium compared to circumcised men^13^. Acceptance rates for MC are high in the United States, Canada, the Middle East, Asian Muslim countries and some African countries, ranging between 20% to 80%^14^. However, MC is not commonly accepted in China. It is estimated that only 2.7% of Chinese males are circumcised, excluding the ethnic Muslim minorities of Hui and Uighur, which have higher rates^15^. The protective effect of MC may have great impact in high prevalence countries where transmission is predominantly heterosexual and MC is not generally practiced^16,17^.

As previously reported, preliminary findings of our cross-sectional survey showed that the willingness of men in Western China to undergo circumcision was only 44.6%^18^. Increasing willingness and medical practice rates of MC is a major public health concern in Western China. Previously we demonstrated that a scale up of MC was more feasible to conduct in high-risk groups such as the migrant population and drug users^18–21^. Nevertheless, no cohort studies have been conducted to assess the effectiveness of MC in preventing HIV infection among the general population in China^19^. To address this gap in knowledge, in the current study we compared three methods for improving the rates of MC acceptance and uptake among men in Western China.

## Methods

### Study design and participants

**Figure 1:**
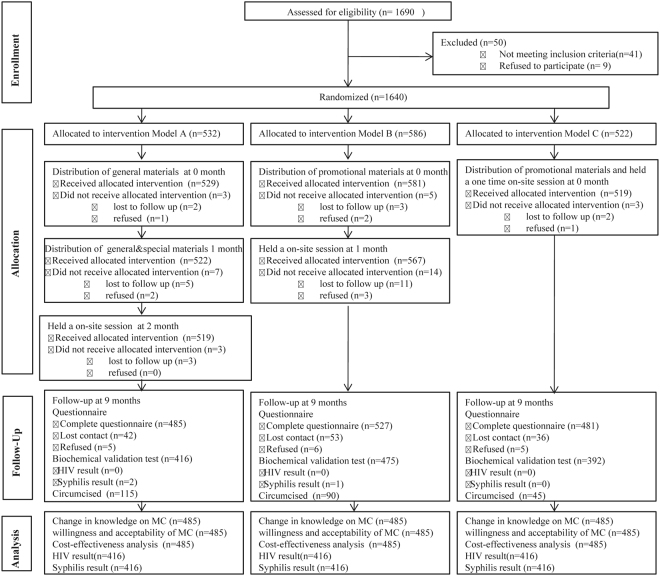
A total of 1690 subjects participated in the initial interview. Of those, 1640 completed the entire interview and were randomly allocated into one of three intervention models. Model A: Delivered all the intervention at 3 stages: distribution of general materials at the initial contact (0 month); distribution of general and special materials within 1 month after the initial contact; held an one-time on-site session that included dissemination of materials and expert- and volunteer-led discussions within 1 month after receiving all the above interventions. Model B: Delivered two intervention performances at 2 stages: distribution of general and special materials at the initial contact (0 month) and held an on-site session that included dissemination of materials and expert- and volunteer-led discussions within 1 month after the initial contact. Model C: Delivered general and special materials and held an one-time on-site session that included dissemination of materials and expert- and volunteer-led discussions at the initial contact (0 month). Changes in knowledge of MC, acceptability of MC, uptake of MC surgery, the cost per acceptance, and HIV/STD infection rate were analyzed at 9-months follow-up visits. For each model, the participants would not have the chance to enter the next intervention if they did not receive allocated intervention, which are included as “lost to follow” or “refused”. Only data generated form participants who completed the entire questionnaire at the 9th month follow-up visit were included in the final analysis.

This study is a focused analysis on one cohort within a larger study evaluating factors influencing Chinese male’s willingness to undergo circumcision (Registration number: ChiCTR-TRC-13004127)^18–21^. Enrollment and 9 month follow-up visits occurred between September 2009 and December 2010. All adult male residents within the selected communities of three Western provinces (Guangxi, Chongqing and Xinjiang) of China were approached for enrollment. The inclusion criteria for participation were: 18–45 years of age, male resident living in the target communities for at least three years, no plans to move from the area in the coming two years, consent to give blood samples for HIV testing, and a negative HIV test result. Participants were excluded from enrollment if they had hearing or speech impairment or had previously undergone MC. All consenting males at each of the three study sites were randomly assigned as a cluster to one of three different intervention models, in which the dissemination of educational materials and expert- and volunteer-led discussions were conducted in one, two, and three stage interventions (Fig. 1).

### Ethics statement

The study was reviewed and approval by the Guangxi Medical University Ethical Review Committee. All study candidates were informed about this study and written informed consent was obtained from every participant prior to enrollment. All participants had the right to withdraw from our study at any time throughout the duration of the study.

### Intervention Models

Our intervention package included a combination of media materials, audio-visual productions, lectures, and experience sharing by peers or medical students. These packages were used previously to investigate the effects of different intervention strategies to promote circumcision among migrant worker populations in the same regions as this study^21^. The different material components of these intervention models are described as follows:

- Factual materials: This component includes general printed materials and audio-visual materials. The printed materials include brochures and orientation pamphlet titled the “Handbook of MC as Protection Against HIV Transmission”, which contain the scientific context of MC, MC and AIDS prevention, benefits of MC in reproductive and sexual health, and pre-operative and post-operative measures. The audio-visual materials are flash animation and scenario educational films, which have the same content as the general printed materials.
- Testimonial materials: This component includes special printed materials and audio-visual materials. The special printed materials introduce MC knowledge by experts and the experiences of medical university students who underwent MC, especially the psychological process of learning about and accepting MC. The special audio-visual materials have the same content as the special printed materials, which were showed by flash animation and scenario educational film.
- In person activities: This component included on-site sessions and discussions led by invited experts and university student volunteers. The theory for this study design was based mainly upon that personal experiences of the experts and volunteers who had previously undergone MC would be a powerful stimulator to promote willingness to accept MC.

We developed three intervention models to evaluate the most effective delivery of all of media materials and on-site sessions and discussions (Fig. 1):

*Model A*: All intervention materials were delivered over three stages: (1) distribution of factual materials at the initial contact (0 month), (2) distribution of general and testimonial materials within 1 month after the initial contact, and (3) a one-time on-site session that included dissemination of additional materials and expert- and volunteer-led discussions within 1 month of receiving the second stage of the intervention.

*Model B*: Two intervention performances delivered over two stages: (1) distribution of factual and testimonial materials at the initial contact (0 month) and (2) an on-site session that included dissemination of materials and expert- and volunteer-led discussions within 1 month after the initial contact.

*Model C*: Factual materials, testimonial materials and a one-time, on-site session that included dissemination of materials and expert- and volunteer-led discussions were delivered upon the initial contact (0 month).

### Interviews and data management

We developed a standardized questionnaire with four sub-sections: (1) demographic characteristics, (2) general knowledge about MC and HIV/AIDS, (3) willingness to accept MC and uptake MC surgery, and (4) reasons to accept or refuse MC. Detailed information regarding the questionnaire has been published previously^18^. Research assistants (RA) received one week of training, which included the details of the study protocol, ethical issues in conducting research, mock interviews using the questionnaire, and collection and storage of blood samples.

After participants provided their written informed consent to participate in the study, RAs conducted the detailed interviews following the approved study protocol. During the follow-up period, participants who were willing to accept MC were referred to the designated hospital with facilities of MC practices to perform the surgery. All circumcision related costs were covered by the project.

### Biological samples

Blood samples were collected from all participants at baseline and at the 9-month follow-up visit. HIV infection was screened by enzyme linked immunosorbent assay (ELISA: Genscreen HIV1/2 version 2, Bio-Rad, France), and confirmed by Western blot (HIV WB: Bio-Merieux-Vitek, St Louis, MO, USA). Samples that were positive by both ELISA and HIV WB were regarded as “positive” and all others as “negative”. A Rapid Plasma Reagin Card Test kit (Macro-Vue RPR: Becton Dickinson, Cockeysville, MD, USA) was used as a serological screen for syphilis.

### Statistical analysis

All the data were double entered into EpiData software (EpiData 3.0 for Windows; The EpiData Association Odense, Denmark) and analyzed using SPSS for windows Version 17.0 (SPSS, Chicago IL, USA). Descriptive analysis was performed on general characteristics and reasons for accepting or refusing MC. Total cost was calculated as the production cost of the promotional materials, service charge, training related expenses, travel related costs, on-site investigation, and intervention costs. Multivariable Cox regression analyses were conducted to compare differences between intervention models, and to estimate relative risk (RR) with 95% confidence interval (CI); adjusted according to demographic and migration characteristics. For statistical analyses of MC uptake, baseline was defined by date of enrollment, and endpoint was defined either by date of uptake of MC surgery for those undergoing surgery or date of final follow-up for those refusing surgery. To compare the incidence of HIV and syphilis between the circumcision and non-circumcision groups, we used the chi-squared test. All statistical tests were two-sided with a significant level of *P* < 0.05.

### Ethical approval

All procedures performed in studies involving human participants were in accordance with the ethical standards of the institutional and national research committee and with the 1964 Helsinki declaration and its later amendments or comparable ethical standards. Informed consent was obtained from all individual participants included in the study.

## Results

### Demographic characteristics

Table 1 shows the demographic characteristics of the participants in our study. A total of 1690 participants participated in the interview and 1640 completed the whole interview (Completion rate: 97%). The 1640 respondents were cluster randomly assigned to one of the three MC-related education intervention groups: Model A (n = 532), Model B (n = 586), and Model C (n = 522). At 9 months, 485 (91.2%), 527 (89.9%) and 481 (92.1%) participants were available for follow-up and provided valid responses to the final questionnaire, respectively. There were no significant differences in age distribution, education level, marital status, and employment status between intervention models (P > 0.05). There were, however, ethnic and religious differences among the participants in the three intervention models at baseline.

**Table 1:** Demographic characteristics of enrolled participants.

Characteristic	Model A (%)	Model B (%)	Model C (%)	Total (%)	*P*
	(N = 532)	(N = 586)	(N = 522)	(N = 1640)	
**Age**					0.066
18–25	289 (54.3)	339 (57.8)	256 (49.0)	884 (53.9)	
25–35	142 (26.7)	143 (24.4)	158 (30.3)	443 (27.0)	
Over35	101 (19.0)	104 (17.7)	108 (20.7)	313 (19.1)	
**Ethnicity**					0.008
Han population	470 (88.3)	547 (93.3)	482 (92.3)	1499 (91.4)	
Other population	62 (11.7)	39 (6.7)	40 (7.7)	141 (8.6)	
**Provinces**					0.120
Guangxi	200 (37.6)	208 (36.1)	194 (40.2)	602 (36.7)	
Chongqing	195 (36.7)	180 (31.3)	181 (34.7)	556 (33.9)	
Xinjiang	137 (25.7)	188 (32.6)	147 (28.2)	472 (28.8)	
**Religion**					0.003
Buddhism	52 (9.8)	32 (5.5)	21 (4.0)	105 (6.4)	
Other religious belief	18 (3.4)	12 (2.0)	9 (1.7)	39 (2.4)	
No religious belief	462 (86.8)	542 (92.5)	429 (94.3)	1496 (91.2)	
**Education level**					0.112
Junior school or below	89 (16.7)	92 (15.7)	106 (20.3)	287 (17.5)	
High school or above	443 (83.3)	494 (84.3)	416 (79.7)	1353 (82.5)	
**Marital status**					0.265
Never married	313 (58.8)	337 (57.5)	301 (57.7)	951 (58.0)	
Married or cohabitation without marriage	215 (40.4)	240 (41.0)	208 (39.8)	663 (40.4)	
Divorced/separated/widowed	4 (0.8)	9 (1.5)	13 (2.5)	26 (1.6)	
**Employment status**					0.253
Employed	490 (92.1)	551 (94.0)	493 (94.4)	1534 (93.5)	
Unemployed	42 (7.9)	35 (6.0)	29 (5.6)	106 (6.5)	

Note: Compared baseline characteristics among Model A, B and C used the chi-squared test.

### Change in knowledge on MC after intervention

Table 2 describes the comparison for the effectiveness of each model in changing participants’ knowledge, attitudes and practices (KAP) regarding MC during baseline and the 9 month follow-up. In all intervention models, knowledge about MC such as reasons for MC and complications of circumcision surgery was substantially increased (P < 0.05). In Model A, there was a higher rate of opinion that MC could enhance sexual activity, and a corresponding lower rate of opinion that MC reduced sexual activity after intervention (P < 0.05) compared to the other intervention models. In Model C, more participants thought MC had no influence on sexual activity, and fewer people did not know the influence at 9 months, compared to baseline (P < 0.05). The two-stage intervention model (Model B) had the greatest increases in general knowledge on MC, potential surgical complications, and the effect of MC on sexual functions by Chi squared test (Table 2).

**Table 2:** Change in knowledge of MC compared between the three intervention models.

Variables	Model A				Model B				Model C			
	Before intervention (%)	After intervention (%)	*x* ^2^	*P*	Before intervention (%)	After intervention (%)	*x* ^2^	*P*	Before intervention (%)	After intervention (%)	*x* ^2^	*P*
	(n = 532)	(n = 485)			(n = 586)	(n = 527)			(n = 522)	(n = 481)		
**Knowledge about surgical reasons**												
Redundant foreskin	365 (68.6)	344 (70.9)	0.6	0.422	408 (69.6)	381 (72.3)	1.0	0.327	371 (71.1)	343 (71.3)	0.007	0.934
Prevention of penile cancer**	169 (31.8)	282 (58.1)	71.5	<0.001	246 (42.0)	324 (61.5)	42.2	<0.001	175 (33.5)	331 (68.8)	124.7	<0.001
Protection against HIV and STDs	147 (27.6)	347 (71.5)	195.9	<0.001	180 (30.7)	399 (75.7)	225.1	<0.001	128 (24.5)	338 (70.3)	210.6	<0.001
Improved female partners’ hygiene*	239 (44.9)	322 (66.4)	47.3	<0.001	316 (53.9)	372 (70.6)	32.6	<0.001	247 (47.3)	303 (63.3)	24.8	<0.001
Enhanced sexual pleasure	173 (32.5)	217 (44.7)	16.0	<0.001	217 (37.0)	233 (44.2)	5.9	0.015	150 (28.7)	183 (38.0)	9.8	0.002
Better penile appearance**	71 (13.3)	106 (21.9)	12.8	<0.001	59 (10.1)	92 (17.5)	12.9	<0.001	38 (7.3)	58 (12.1)	6.6	0.010
Traditional or religious reason	3 (0.6)	12 (2.5)	6.4	0.012	0	12 (2.3)	13.5	<0.001	2 (0.4)	6 (1.2)	2.4	0.124
**Knowledge about surgical complications**												
Pain	204 (38.32)	304 (62.7)	60.0	<0.001	250 (42.7)	361 (68.5)	74.8	<0.001	189 (36.2)	302 (62.8)	70.8	<0.001
Bleeding**	121 (22.7)	238 (49.1)	77.0	<0.001	275 (46.9)	329 (62.4)	26.9	<0.001	143 (51.5)	313 (65.1)	143.3	<0.001
Infection**	227 (42.7)	242 (49.9)	5.3	0.021	166 (28.3)	289 (54.8)	80.7	<0.001	269 (27.4)	366 (76.1)	65.0	<0.001
Don’t know**	126 (23.7)	88 (18.1)	4.7	0.030	138 (23.5)	62 (11.8)	28.0	<0.001	112 (21.5)	33 (6.9)	43.1	<0.001
**Influence to sexual activity**												
Enhanced**	144 (27.1)	237 (48.9)	51.5	<0.001	209 (35.7)	218 (41.6)	3.8	0.051	148 (28.4)	156 (32.4)	2.0	0.160
Reduced**	47 (8.8)	18 (3.7)	11.1	0.001	43 (7.3)	27 (5.1)	2.3	0.129	58 (11.1)	43 (8.9)	1.3	0.254
Has no influence*	119 (22.4)	118 (24.3)	0.1	0.783	113 (19.3)	123 (23.3)	2.7	0.098	116 (22.2)	146 (30.4)	8.6	0.003
Don’t know*	125 (23.5)	112 (23.1)	0.02	0.879	149 (36.0)	159 (30.2)	3.1	0.077	183 (35.1)	136 (28.3)	5.3	0.021

Note: Compare to changes in knowledge on circumcision of three models for scaling up MC after education intervention among three models. *P < 0.05; **P < 0.01.

### Change in willingness to accept and uptake MC surgery

After the three interventions, there were significant differences in participant willingness to accept and uptake MC surgery (P < 0.05) (Table 3). While willingness to accept MC increased after intervention in all three models, increasing from 44.7% (238/532), 50.9% (298/586), and 39.3% (205/522) to 52.6% (255/485), 67.0% (353/527), and 45.5% (219/481) in Model A, Model B and Model C, respectively (P < 0.05), Model B had the largest increase in willingness to accept MC after the intervention.

**Table 3:** Change in willingness to accept MC surgery after intervention.

Variables	Willingness to accept MC			
	Before intervention (%)	After intervention (%)	*x* ^2^	*P*
Model A	44.7 (238/532)	52.6 (255/485)	6.2	0.012
Model B	50.9 (298/586)	67.0 (353/527)	31.6	<0.001
Model C	39.3 (205/522)	45.5 (219/481)	26.9	<0.001
*x* ^2^	15.013	49.141	—	—
*P*	<0.001	<0.001	—	—
Total	45.2 (741/1640)	55.4 (827/1093)	32.6	<0.001

As for improving the participants’ uptake of MC surgery, surgery rate increased sharply among participants in Model A (23.7%; 115/485) compared to participants in Model B (17.1%; 90/527) and Model C (9.4%; 45/481) (P < 0.05). Cox-regression analysis revealed that uptake of MC surgery was statistically different among the three intervention models, with the three-stage model (Model A) being the most effective (Table 4). Comparing Model A or Model B to Model C, RRs were 2.4 (95% CI, 1.5–3.8, P < 0.01), and 2.2 (95% CI, 1.3–3.6, P < 0.01), respectively, indicating that Model A and Model B were approximately twice as effective as Model C. Differences between Model A and Model B were not statistically significant (*P* = 0.655).

**Table 4:** Change in uptake MC surgery after intervention and Cox-regression analysis.

Variables	Uptake of MC surgery				Cox-regression of uptake of MC		
	Before intervention (%)	After intervention (%)	*x* ^2^	*P*	RR*	95% CI	*P*
Model A	0	23.7 (115/485)	142.2	<0.001	2.4	1.5–3.8	<0.001
Model B	0	17.1 (90/527)	108.9	<0.001	2.2	1.3–3.6	0.002
Model C	0	9.4 (45/481)	51.1	<0.001	1	—	—
*x* ^2^	—	35.765	—	—	—	—	—
*P*	—	0.000	—	—	—	—	—
Total	0	22.9 (250/1493)	298.4	<0.001	—	—	—

Note: RR*, the rate of MC acceptability that Model A and Model B compared to Model C, respectively. Cox regression model adjusting for demographic (age, ethnicity, marital status and education) and regions, estimated RR (relative risk) with 95% CI (confidence interval). Defining the baseline time as the starting point, the endpoint was either acceptance of MC surgery or, for uncircumcised participants, the end of the second follow-up session.

### Cost-effectiveness analysis

The input cost was defined as the total cost of the model divided by the number of recipients who received the model, while the cost effectiveness was assessed by dividing the total cost with the number of participants who underwent MC surgery. Model B required the lowest cost per circumcision, with each case costing only 574RMB (US$92). Model C was most expensive, requiring 698RMB (US$110) per case, while Model A cost 623RMB per circumcision (US$100).

### HIV/STD incidence

Throughout the duration of this study, none of the participants acquired HIV infection. Syphilis incidences were 0 per 100 person-years in the MC group compared to the 0.28 per 100 person-years in the non-MC group (*P* = 0.936 > 0.05).

## Discussion

Heterosexual intercourse has become a dominant route of HIV transmission, accounting for nearly 70–80% of infections in Western China in 2016^1^. Additionally, in this region, safe sex practices are not a cultural norm and access to condoms is often unreliable. Therefore, promoting MC among China’s male population may therefore have important implications for reduction in HIV prevalence in Western China^1,22^. Compared to other means of partial protection such as condom use, MC is a one-time intervention with lifelong benefits. Our study showed that the knowledge about MC among the general population including reasons for MC and complications of circumcision surgery substantially increased after receiving educational interventions, particularly increasing knowledge on the role of MC in preventing HIV/AIDS and STD. The two-stage intervention (Model B) was the most effective model at improving participants’ willingness to accept MC, while the three-stage intervention (Model A) was the most effective at increasing actual uptake of MC surgery.

There were significant differences between acceptability of MC and uptake of MC surgery as a result of each intervention model. More than half of the participants were willing to accept MC, suggesting that interventions to scale up circumcision among the general population have a high likelihood of success. This finding was similar to previous studies conducted amongst migrant populations and bisexual men who have sex with men (MSM)^21,23^. In a previous study assessing these same intervention strategies on the MSM population, willingness to undergo circumcision increased from 8.1% to 35.1% after consulting^23^. Despite the relatively high reported rate of willingness to accept MC (55.4%), in our study only 22.9% participants actually underwent the surgery after intervention, and thus, willingness and undertake of MC surgery were not strongly correlated. These data suggest that self-reported attitudes towards MC is not strongly correlated with actual behavior in the general male population, which is similar to our previous study conducted among the migrant worker population in this same region of western China^21^.

Compared to some regions of Sub-Saharan Africa, our results showed a much lower acceptance rate of MC amongst the general population, likely due to a lack of history and cultural norms regarding MC^24^. In pooled data from thirteen studies encompassing nine countries in Sub-Sahara Africa, the median proportion of uncircumcised men willing to become circumcised was 65% (range 29–87%)^25^. A similar study, based in South Africa, showed that through a local effort to scale up MC, 39.1% (14,011/35,851) male participants accepted MC over a 12-months follow-up period, equivalent to 701 people per month^12^.

A Cox’s proportional hazards regression model of the data in our study suggested that Model A or Model B had significantly greater effects than Model C on improving knowledge of and willingness to accept MC. In addition, Model A significantly increased the proportion of participants who actually underwent MC surgery. These results indicate that a multi-staged intervention that gradually reinforced knowledge through educational materials and volunteer and expert-led discussions has a much greater effective on promoting behavior change of male participants in Western China. The involvement of peers, partners, and medical college students likely played an important role in encouraging participants to retain and internalize knowledge on MC and to take action on seeking MC surgery^26,27^.

While this study reveals some important information regarding the effectiveness of educational interventions on increasing acceptance and uptake of MC, there were several limitations to this study. First, the 9-month follow-up time of our program was very short, and thus greatly limited our ability to accurately assess the impact of MC on acquisition of HIV or other STIs. As a result, during the follow-up period of our study, no participants acquired HIV infection. In addition, the incidence of syphilis was very low, and therefore there were no significant findings between the MC group and non-MC groups. Furthermore, one possible reason for our results were not strongly consistent with other studies because of the huge difference in HIV prevalence between China (~0.06%) and other countries such as Kenya (>2.5%)^1,28^. A randomized controlled trial in Kisumu, Kenya showed that the 2-year HIV incidence rates were 2.1% (95% CI: 1.2–3.0) in the circumcision group and 4.2% (95% CI: 3.0–5.4) in the control group^11,29^. The results in our study need to be confirmed by further research with a longer follow-up time after MC to assess the incidence rates of HIV and other STIs. Additionally, measurement of the knowledge on MC relied on self-report and was likely impacted by social desirability, which could result in under or over-reporting. Nevertheless, there was no a priori expectation for the direction in which this might occur, nor any suggestion that this should differ between different intervention models^30^. Finally, in order to prevent contamination between the different interventions models within the same site, we performed cluster randomization rather than individual randomization, in which the entire group of participants at each study site was allocated randomly into one of the three intervention models.

We believe that the conditions for large HIV epidemics are ripe within this demographic of Western China, and investigations into effective, feasible, and sustainable interventions should be of high priority. Combined with the large increase in transmission attributable to heterosexual intercourse, there is a significant need for interventions focused on prevention of sexual transmission in this region.
